# Safety of intravenous iodinated contrast medium injection in rabbits undergoing conscious computed tomography

**DOI:** 10.1002/vro2.31

**Published:** 2022-03-04

**Authors:** Ingrid Isaac, Jenna Richardson, Tiziana Liuti, Maurizio Longo

**Affiliations:** ^1^ Royal (Dick) School of Veterinary Studies and Roslin Institute The University of Edinburgh Roslin UK; ^2^ Department of Veterinary Medicine Veterinary Teaching Hospital University of Milan Lodi Italy

## Abstract

**Background:**

Contrast media in CT is widely used in dogs and cats to provide superior tissue delineation and increase the diagnostic capabilities. These contrast‐enhanced imaging techniques are gaining popularity in rabbits; published studies reporting the safety of doing so are lacking.

**Methods:**

This retrospective observational study aimed to determine the incidence of adverse events following the intravenous administration of iodinated non‐ionic contrast medium in 350 rabbits. The medical records of this subset of rabbits admitted between January 2009 and November 2018, that underwent CT examination and received intravenous contrast media, were evaluated.

**Results:**

From the 350 rabbits, 342 rabbits were still alive 7 days after the scan. A total of eight rabbits died within 7 days of the scan, seven of which within the first 24 h. All deaths were presumed to be sequelae to the diseases they were being investigated for and not thought to be related to the administration of intravenous contrast medium.

**Conclusions:**

The results indicated that the use of intravenous non‐iodinated contrast medium was well tolerated and safe in rabbits undergoing conscious CT examination.

## INTRODUCTION

The popularity of domestic rabbits (*Oryctolagus cuniculus*) as companion animals has increased in recent years,^1^ resulting in an increased influx of these animals into veterinary hospitals and a greater demand for diagnostic imaging investigations.

Among the most common health concerns in rabbits are dental abnormalities. Due to the eruption and continuous growth of their teeth, inadequate dietary fibre can lead to dental overgrowth, facial abscesses, periodontal and nasolacrimal duct diseases.^2^ Suboptimal diet and inadequate husbandry can be underlying causes for illness in rabbits. The increasing longevity of these animals has contributed to a higher incidence of chronic and age‐related diseases within the population, which may result in higher susceptibility to infection and immunosuppressive diseases.^3^ Additionally, as prey species, rabbits hide signs of illness and often appear to present with an acute condition that results from multiple subacute problems. Obtaining a cross‐sectional imaging study of the entire animal (whole body), with high‐resolution and multi‐planar reconstructions, within a short scan time, makes CT one of the most useful imaging modalities among veterinary surgeons dealing with these animals. Another advantage of CT is that it can be performed on a conscious rabbit, by placing the animal in a purpose‐built restraint device, such as the VetMouseTrap (Universal Medical Systems Inc., Solon, USA), avoiding the potential anaesthesia‐related complications associated with the anatomical and physiological species‐specific features of rabbits, which include gastrointestinal stasis.^4^ Furthermore, the VetMouseTrap can serve as an oxygen chamber, opening up investigatory options of advanced imaging for dyspnoeic cases.

**FIGURE 1:**
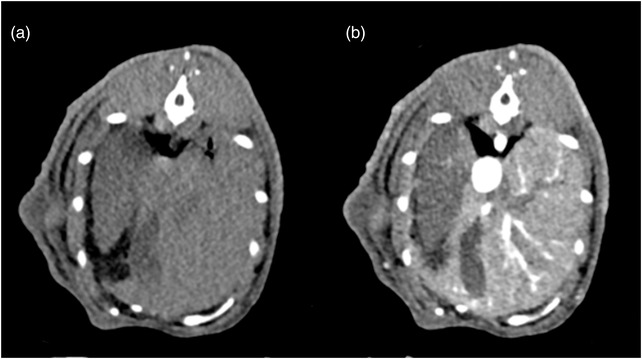
Pre‐contrast (a) and post‐contrast (b) computed tomographic transverse images of the cranial abdomen at the level of the liver (helical mode, slice thickness 1.5 mm, soft tissue algorithm). The right lateral hepatic lobe (arrowheads) is hypodense (a) and non‐contrast enhancing (b) indicative of a liver lobe torsion

**FIGURE 2:**
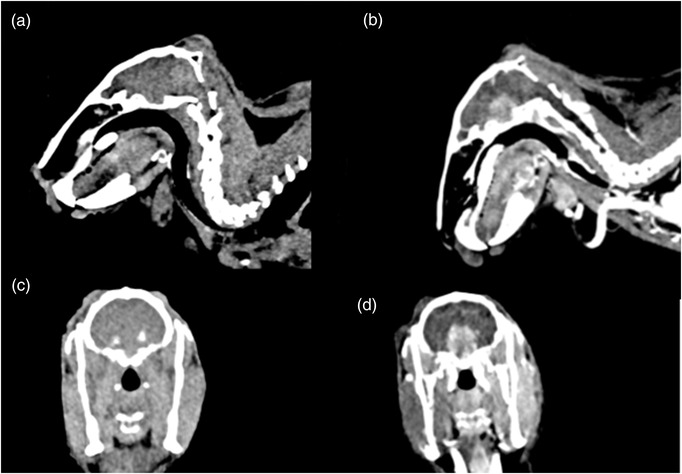
Pre‐ and post‐contrast computed tomographic sagittal (a and b) and transverse images (c and d) of the head of a rabbit (helical mode, slice thickness 1.5 mm, soft tissue algorithm). On the post‐contrast examinations (b and d) a large, well‐defined, oval and strongly contrast enhancing suprasellar soft tissue mass is visible compatible with pituitary neoplasia

In both veterinary and human medicine, contrast media is an integral part of the CT examination. Its purpose is to enhance the ability to appreciate anatomical detail. In rabbits, definition of the soft tissue structures within the abdomen can be challenging. The use of intravenous contrast medium improves the level of detail and highlights conditions that are frequently diagnosed in rabbits such as gastrointestinal pathology including sacculitis and appendicitis,^5^ liver lobe torsions (Figure 1a,b), neoplasia and granulomatous diseases. It improves the level of detail in evaluation of ear base swellings,^6^ dental abscesses and intracranial neoplasia (Figure 2a–e).

Contrast enhancing can be thought of in two phases—the first is a direct result of blood flow and the second reflects vascular permeability, as the contrast agents freely diffuse across the endothelial walls.^7^ The contrast agents used in CT are iodine based and may be ionic or non‐ionic. The ionic agents (1900–2100 mOsm/kg) are much more hypertonic than non‐ionic agents (290–900 mOsm/kg) or blood plasma (median measured osmolality for dogs is 302 mOsm/kg,^8^ for cats is 317 mOsm/kg^9^ and for rabbits is 301 mOsm/kg),^10^ therefore the likelihood of creating an adverse reaction to its administration is higher.^7^ The use of non‐ionic agents is thus preferred.

The adverse reactions to contrast media administration have been investigated and documented in human medicine and are classified as acute or delayed, based on their time of onset.^11^ The acute reactions occur within the first hour and the delayed reactions begin 1 h to 7 days after the contrast administration.^12^ In humans adverse reactions can be subclassified according to the severity of the symptoms and clinical features as severe, moderate or mild. Severe and moderate reactions can be life threatening and require immediate management. These include profound bronchospasm, dyspnoea, sudden hypotension, angioedema, convulsions, loss of consciousness, cardiovascular shock, arrythmia and cardiorespiratory arrest.^11^, ^13^ Mild reactions do not require specific treatment, are self‐limiting and of short duration and typically affect the skin.^14^, ^15^, ^16^ The risk of adverse reactions has decreased over time with the preferred use of non‐ionic low‐osmolality formulations instead of the ionic, high‐osmolality agents.^11^ The most commonly reported adverse reactions (70%) in humans to the non‐ionic agents are dermatological and are associated with pruritus and urticaria.^11^, ^12^

In veterinary medicine, there are limited reports on reactions to the use of contrast agents. The mild and moderate side effects reported in dogs and cats include twitching, changes in heart rate, blood pressure, vomiting and facial oedema.^17^, ^18^, ^19^ Severe, anaphylactic or anaphylactoid reactions have been reported in two dogs following administration of an ionic contrast agent and one cat receiving a non‐ionic formulation. The two dogs were reported to have immediate severe changes in heart rate and systolic blood pressure leading to cardiovascular collapse^18^ and the one cat had signs of immediate bradycardia and a drop in the pulse rate, resulting in cardiac arrest.^20^

Despite the associated risks with the administration of iodinated contrast agents, it is widely recognised in both the human and veterinary fields, that the benefits of its use exceed the possible complications.

The purpose of the present study was to determine the incidence of adverse events following the administration of intravenous iodinated non‐ionic contrast media in rabbits, which to the best of the authors’ knowledge, has not yet been reported.

## MATERIALS AND METHODS

### Study design

A retrospective analysis was performed to review the medical records database of the Hospital for Small Animals at the Royal (Dick) School of Veterinary studies, for pet rabbits that underwent a CT study for diagnostic purposes between January 2009 and November 2018. This study was approved by the Ethical Committee at the University of Edinburgh (Veterinary Ethics and Welfare Committee reference 70.19).

### Clinical data

For rabbits meeting inclusion criteria, the following medical record data were recorded: age at time of imaging, breed, sex, weight at time of imaging, date of imaging, contrast medium administration and if deceased within 7 days after the CT scan, cause of death and results of a postmortem examination if available.

### Image acquisition

Images were acquired with the use of two different CT scanners: a four‐row Multi‐detector row computed tomography (MDCT) unit (Somatom Volume Zoom, Siemens, Germany) from January 2009 to October 2016 and a 64‐row MDCT unit (Somatom Definition AS Siemens, Erlangen, Germany) from October 2016 to November 2018. The cross‐sectional images were acquired on conscious rabbits, restrained within a device, the VetMouseTrap. Scan settings included a pitch of 1.5, tube potential of 120 kVp, reference tube current of 160 mA, slice thickness of 1.5 mm, matrix 512 × 512, and reconstruction with low and high‐frequency algorithms. A bolus of 740 mg iodine/kg of non‐ionic iodinated contrast medium (Iopamiro 370 mg/ml, Bracco, Manno, Switzerland and Iomeron 350 mg/ml, Bracco) was injected through an angiocatheter placed within the marginal auricular vein. The rabbits examined from 2009 to 2015, had the contrast agent administered manually, followed by 1.5 ml of saline solution flush and post‐contrast images were acquired within 1 min after the contrast administration. From October 2016 onwards, the contrast was administered via a power injector pump (CT Exprès™ Injector Unit, Bracco Injeneering S.A., Lausanne, Switzerland) with a flow velocity of 0.8 ml/s and post‐contrast images were acquired within 18 s of contrast administration.

## RESULTS

### Study population

In total, 350 rabbits were included in the study and received intravenous contrast medium. All of the rabbits were scanned consciously in a purpose‐built restraint device. A variety of breeds were represented: dwarf lop, mini lop, lionhead, Dutch, French lop and mini rex. The age range of the rabbits varied between 1 and 10 years (1 year—*N* = 26; 2 years—*N* = 41; 3 years—*N* = 55; 4 years—*N* = 44; 5 years—*N* = 47; 6 years—*N* = 45; 7 years—*N* = 44; 8 years—*N* = 29; 9 years—*N* = 13; 10 years—*N* = 6) with a mean age of 4.8 years. There was no significant sex bias with 180 males (147 neutered and 33 entire) and 170 females (139 neutered and 31 entire).

The contrast medium administration was well tolerated in all the individuals (*N* = 350), with no registered immediate adverse reactions or sudden death. From the 350 rabbits, 40 (≅11%) were euthanised within 7 days of the CT scan due to poor prognosis based on clinical deterioration and/or imaging findings which included neoplasia, severe dental disease, complex femoral fractures and severe spinal disease.

Eight rabbits (≅2%) died within 7 days of the intravenous administration of the contrast medium. Of these eight rabbits, no deaths were believed to be linked directly to the administration of the contrast medium. Rabbits A–G died within 24 h of contrast administration, while Rabbit H died 6 days later. Rabbit A, diagnosed with a liver lobe torsion, died in recovery from emergency surgery for a haemo‐abdomen and liver lobe resection. Haemodynamic instability was believed to be a main factor. Rabbit B presented with non‐specific signs of gastrointestinal stasis and was referred for advanced imaging. Postmortem examination led to a diagnosis of rabbit haemorrhagic disease virus strain 2 (RHDV‐2). Rabbit C underwent CT evaluation for investigation of a suspected retrobulbar abscess. Death resulted after iatrogenic administration of penicillin by an intravenous route. Rabbits D and E both died following complications during a general anaesthetic, performed after the CT studies. Rabbit D underwent a CT scan to investigate the presence of gross metastatic disease, pre‐neutering, following palpation of a uterine mass. There were no gross signs of metastases and the ovariohysterectomy went ahead the following day, however the animal deteriorated when recovering from the general anaesthesia. Rabbit E underwent successful resuscitation following cardiac arrest during a general anaesthetic for a dental procedure. After recovery, the rabbit underwent a conscious CT evaluation and was diagnosed with lung pathology and serosanguinous pleural effusion, which was drained under ultrasound guidance. Respiratory signs continued to worsen within 24 h and the animal collapsed for a second time, without successful resuscitation. Rabbits F and G both presented with neurological signs and CT was performed to evaluate the brain, middle and internal ears. With no structural abnormalities noted, a presumptive diagnosis of *Encephalitozoon cuniculi* was given. Both animals continued to deteriorate with hindlimb paresis, reducing responsiveness and, in the case of Rabbit F, opisthotonos, before death. The final rabbit, Rabbit H, died following deterioration of a non‐surgically treated, colonic obstruction. The death occurred 6 days post‐CT assessment for intermittent gastrointestinal stasis, where at that time‐point, no obstruction had been present. The cause of death was confirmed at postmortem examination.

The rabbits examined between 2009 and 2015, received the intravenous contrast media administered manually (*N* = 76) and the rabbits scanned between 2016 and 2018 (*N* = 274), received the contrast via power injector pump. Two of the 76 rabbits (≅2.6%) and five of the 274 rabbits (≅2%) died acutely, within 24 h after the CT examination. One of the 76 rabbits (≅1.3%) died 6 days after CT examination, due to colonic obstruction, confirmed on postmortem examination.

Twenty‐three rabbits underwent repeated scans over time: 15 had a CT scan repeated once, five had a CT scan twice, two had CT scan three times and one rabbit was CT scanned five times. All of the repeated scans occurred with at least 1 month gap between them. None of these rabbits had reported adverse reactions from the use of contrast media on their medical records, neither died within 7 days of the CT examinations.

## DISCUSSION

The adverse events following intravenous contrast administration in humans can be classified as immediate or delayed (from 1 h up until 7 days post‐contrast administration).^11^ In the present study, no immediate adverse reactions or sudden death were detected in the rabbits receiving contrast media. Even though seven rabbits died within the first 24 h following contrast administration, their deaths were believed to have been precipitated by their clinical conditions. From the eight rabbits that died within 7 days of receiving contrast, two suffered complications on recovery from surgery and two others presented with severe neurological conditions raising a strong concern over *E. cuniculi* infection. One died following erroneous administration of penicillin. Two other rabbits had postmortem confirmation of disease. Finally, one of the rabbits had decompensated on recovery from liver lobe resection and haemodynamic compromise.

With the development of non‐ionic low‐osmolar iodinated contrast media, the incidence of adverse reactions in human patients has greatly diminished and about 70% of the reported adverse reactions are dermatological and self‐limiting, with manifestations of pruritus and urticaria.^11^, ^13^ The formerly used ionic contrast agents were largely associated with contrast‐induced acute kidney nephropathy (CIN), with high morbidity and mortality rates.^21^ The occurrence of CIN in dogs following contrast media administration has been documented, with a proposed incidence of 7.6%; but, due to the small population of this one study, a true incidence and clinical relevance of this syndrome could not be established in a general population of dogs.^22^ The risk of CIN is expected to be higher in animals with already compromised renal function.

Chronic kidney disease is the most well‐known risk factor for the use of iodinated ionic contrast agents in humans, yet other recognised conditions can increase the risk of adverse reactions, including diabetes, cardiac disease (particularly coronary artery disease) and dehydration.^11^ While there is no available data in the veterinary literature regarding the influence of these factors in rabbits, it is likely that the presence of cardiac and/or renal disease could increase the risk of contrast reactions, especially if ionic agents are used.

In humans, asthma, allergic conditions and atopic skin disease appear to correlate with an increased risk of contrast medium‐related reactions.^11,^, ^23^ The incidence and prevalence of these diseases in rabbits is low, with no published case studies of asthma or atopic dermatitis in rabbits. Correlations with this and contrast reactions therefore requires further investigation in veterinary medicine.

Repeated use of contrast media has been documented in human literature as a possible predisposing factor to hypersensitivity reactions; this is a risk that may be higher when ionic contrast agents are used (17%–35%) in comparison to the risk of using non‐ionic agents (5%).^14^ In veterinary medicine, a study performed in healthy dogs that received repeated doses of contrast medium (non‐ionic, iohexol) within 6–8 weeks reported no significant clinical effects from this repeated use.^24^ In the present study, 23 rabbits underwent repeated CT examinations with use of contrast media as part of the protocol, none of them demonstrated adverse clinical reactions from repeated use.

Limitations of the present study include its retrospective nature and the lack of data such as pre‐ and post‐contrast administration serum biochemical analysis, haematology, heart rate and blood pressure values. In one study,^19^ the most commonly reported adverse reactions to contrast media administration (iohexol and gadolinium), in cats and dogs, were classified as moderate, with alteration of the haemodynamic and/or respiratory parameters. Rabbits were scanned conscious with use of a restraint device. This did not permit the use of continuous monitoring equipment, for example Doppler or electrocardiogram for heart rate, capnography for respiration, rectal thermometer for temperature. As prey species, rabbits can be sensitive to handling, assessment of vital parameters in a continuous way is neither practical nor recommended, due to the risk of additional stress of these monitoring methods. The included rabbits demonstrate an unintended selection bias, as these were clinically unwell, admitted for non‐elective investigations. Healthy animals would be expected to experience even fewer complications.

The concentration of the contrast agent used was the same for all the rabbits. However, there was a bimodal administration of the contrast, performed both manually and via a pressure injector pump, which also poses as a limitation for the present study. The rate of the bolus injection should be standardised to allow the appropriate enhancement pattern of the various organs and potential lesions. It has been suggested that higher speed injections are linked to higher rates of contrast reactions in humans.^15^ According to our records, from the 76 cases that received manual administration of contrast, two died acutely (≅2.6%) within 24 h after the CT study and from the 274 cases that received contrast via the power injector, five suddenly died (≅2%) within the same time period. Even though a direct comparison between the flow rates used manually and via injector could not be performed retrospectively, it is believed that this variation played a minimal influence on the obtained results, as in both cases the number of sudden deaths was low and was not clinically believed to be related to contrast administration. Ultimately, postmortem examinations were only performed in two of the eight deceased rabbits to establish a definitive cause of death.

The results of our study suggest that the intravenous administration of iodinated non‐ionic contrast media is well tolerated and safe in rabbits undergoing conscious CT examination. The incidence of adverse events and mortality within 7 days after the administration of the contrast agent in rabbits was low. The risk of developing adverse events following the administration of non‐ionic contrast media may be higher in rabbits with cardiovascular and renal diseases; further prospective studies are needed to evaluate the degree of correlation.

## CONFLICTS OF INTEREST

The authors declare they have no conflicts of interest.

## ETHICS STATEMENT

The authors confirm that the ethical policies of the journal, as noted on the journal's author guidelines page, have been adhered to. The research was approved by the Ethical Committee at the University of Edinburgh (Veterinary Ethics and Welfare Committee reference 70.19).

## Data Availability

The data that support the findings of this study are available from the corresponding author upon reasonable request. The data will be anonymised and made available on reasonable request.
